# Maraviroc enhances Bortezomib sensitivity in multiple myeloma by inhibiting M2 macrophage polarization via PI3K/AKT/RhoA signaling pathway in macrophages

**DOI:** 10.1186/s13008-025-00145-1

**Published:** 2025-02-14

**Authors:** Huiye Yang, Yuchan He, Fujun Qu, Jie Zhu, Liyuan Deng, Fang Jiang, Xianyi Wu, Yixuan Chen, Ali Kashif, Xiaotao Wang

**Affiliations:** 1 Department of Hematology, Affiliated Hospital of Guilin Medicial University, Lequn Road 15#, Guilin City, Guangx 541000 China; 2 Department of Hematology, The Second Affiliated Hospital of Guilin Medical University, Guilin, 541199 China

**Keywords:** Multiple myeloma, CCL3/CCR5, PI3K/AKT/RhoA, M2 polarization, Tumor microenvironment

## Abstract

**Background:**

Multiple myeloma (MM) is a malignancy where drug resistance often leads to relapse or refractory disease. Chemokine receptor 5 (CCR5) has emerged as a novel therapeutic target. However, the role of CCR5-antagonist Maraviroc (MVC) in M2 macrophage polarization and its potential to enhance Bortezomib sensitivity in MM has not been fully explored.

**Methods:**

We used human bone marrow samples, RPMI 8226 cells, and THP-1 monocytes to investigate CCL3/CCR5 axis. ELISA measured CCL3/CCR5 levels. Knockdown/overexpression vectors modulated expression. Cell proliferation, apoptosis, and macrophage polarization were assessed using CCK8, flow cytometry, and transwell assays. QRT-PCR analyzed CCL3 expression, and western blotting examined PI3K/AKT/RhoA signaling. CCR5 was targeted via siRNAs or MVC. NOD/SCID mouse model evaluated CCL3/CCR5 effects on macrophage polarization and MVC’s impact on Bortezomib efficacy.

**Results:**

CCL3, CCR5, and M2 macrophage markers are upregulated in MM patients, with CCL3/CCR5 expression correlating with M2 macrophage polarization. Myeloma-secreted CCL3 and paracrine CCR5 significantly promoted M2 macrophage polarization by activating PI3K/AKT/RhoA signaling, which in turn enhanced myeloma proliferation, inhibited apoptosis, and reduced Bortezomib sensitivity. MVC inhibited M2 macrophage polarization and improved Bortezomib sensitivity in vitro and xenograft mouse myeloma models.

**Conclusions:**

MVC reduced macrophage polarization and enhanced Bortezomib sensitivity in MM cells.

**Supplementary Information:**

The online version contains supplementary material available at 10.1186/s13008-025-00145-1.

## Introduction

Multiple myeloma (MM), which ranks as the second most common hematological malignancy in the bone marrow microenvironment (BMME), is characterized by extensive heterogeneity and clonal evolution [1, 2]. Over the past decades, with emerging treatments such as proteasome inhibitors, immunomodulatory drugs, and [1] CD38 antagonists, the prognosis for MM patients has considerably improved [3]. However, resistance to these drugs often leads to relapsed or refractory disease [4]. Given that the tumor microenvironment significantly influences progression, antitumor immunity, and drug resistance in MM, the supportive BMME is considered one of the main contributors to MM resistance and recurrence [5]. An increasing volume of research suggests that MM cells interact with bone marrow (BM) immune cells to induce drug resistance via cell-cell contact and secretion of cytokines, receptors, adhesion molecules, and chemokines, suggesting that these factors participate in the progression and drug resistance of MM [6]. Therefore, increased understanding of the pathological mechanism of interactions between MM cells and immune cells in the BMME may provide insights for the development of new therapies.

A previous study from our laboratory demonstrated that macrophage inflammatory protein 1-alpha (CCL3) mediates tumor growth, progression, and bone destruction in MM [7]. CCL3 and its receptors, CCR1 and CCR5, are known to play a significant role in the progression of bone disease by promoting the proliferation of MM cells and modulating osteoclast differentiation [8]. CCL3 binds to CCR1 to protect MM cells from apoptosis induced by melphalan and bortezomib, thereby leading to chemoresistance [9, 10]. Additionally, the CCL3/CCR5 axis is crucial in the malignant progression of a variety of tumors and the regulation of macrophage polarization [11]. Recent research indicates that lenalidomide and pomalidomide may reduce CCR5 expression levels in peripheral blood mononuclear cells (PBMCs), consequently combating MM cells and ameliorating disease progression [12]. However, the precise function of CCR5 in the MM microenvironment remains unclear, and its potential utility as a therapeutic target for MM has yet to be demonstrated.

Macrophages infiltrating into the tumor microenvironment (TME), called tumor-associated macrophages (TAMs), constitute the most abundant immune cell type [13]. TAMs exhibit high plasticity and are categorized into two polarization subtypes: classically activated (M1-type macrophages, exhibiting antitumor activity) and alternatively activated (M2-type macrophages, displaying pro-tumor activity). M1-type macrophages express the surface markers CD80 and CD86, while the main surface markers of M2-type macrophages are CD163 and CD206 [14]. TAMs originate from circulating monocytes after recruitment to the TME by cancer cells and progressively acquire pro-tumor properties [15]. In the MM BMME, the TAMs are predominantly M2 macrophages, which increase in number with disease progression. M2-type macrophages have been shown to promote plasma cell proliferation and angiogenesis, further supporting MM immune evasion [16]. In the context of novel therapeutic agents, the level of TAMs is emerging as an independent prognostic factor for poor outcomes, with a higher number of TAMs corresponding to a lower rate of complete remission, acquired drug resistance, and reduced progression-free, and overall survival [17]. Therefore, the targeting of TAMs is recognized as a promising approach in cancer therapy, highlighting their importance for the development of effective treatment and prognosis strategies for MM.

In previous studies, we observed that samples from active MM patients exhibit activation of the PI3K/AKT and RhoA/ROCK1 signaling pathways, which are known to facilitate tumor cell proliferation [18, 19]. Moreover, in recent years, patients treated with the CCR5 ligand, maraviroc (MVC), have shown a deceleration in tumor development, especially for metastatic colorectal cancer. In the PICCASSO phase I trial, the CCR5 antagonist demonstrated anti-colorectal-tumor activity and reduced macrophage infiltration. However, it is unclear whether MVC has anti-myeloma effects. In this study, we first comprehensively demonstrate that the polarization of M2 macrophages accelerates disease progression by utilizing autocrine CCL3 and paracrine CCR5, which subsequently activates the PI3K/AKT/RhoA signaling pathway within the BMME. Secondly, we validate that targeting the CCR5 axis can inhibit M2 macrophage polarization and enhance sensitivity to anti-myeloma drugs, thereby potentially improving the prognosis for patients with myeloma.

## Results

### Elevated CCL3/CCR5 expression in the BM of MM patients is associated with increased M2 macrophage CD206 expression score

The clinical characteristics of the patients in this study are summarized in Table 1. Spearman’s correlation tests revealed a high expression score for the M2 macrophage marker CD206 was positively associated with high tumor burden, relapse, and cytogenetic abnormalities (*p* < 0.05). Specifically, the median age of the 37 MM patients in the test group was 58 years (range, 43–76 years). Among them, 20 (54.1%) were male and 17 (45.9%) were female. Additionally, the control group consisted of 15 healthy volunteers with a median age of 32 years (range, 24–52 years), including 10 males (66.7%) and 5 females (33.3%).

**Table 1:** Correlation of CCL3 and CCR5 expression with clinical and lab parameters in MM patients

Patient’s parameters	Total, median (range) (*n* = 37)	CD206 expression level		*P* value
		High, % (*n* = 21)	Low, % (*n* = 6)	
male (/female)	20/17	56.76	16.21	0.893
Age, ≥ 65 years	58 (43–76)	13.51	10.8`	0.818
ISS stage	III (II, III)	32.43	28.57	0.613
LDH, ≥ 230 IU/L	258 (138–639)	21.62	37.83	0.116
M-protein(g/l)	32.6(11.89–54.67)	48.65	18.91	0.004**
Relapase	18	40.54	8.11	0.002**
Cytogenetic abnormalities	18	36.8	15.8	0.033*

Abbreviations: MM, multiple myeloma; LDH, lactate dehydrogenase; ASPC, bone marrow aspiration plasma cells

**Fig. 1:**
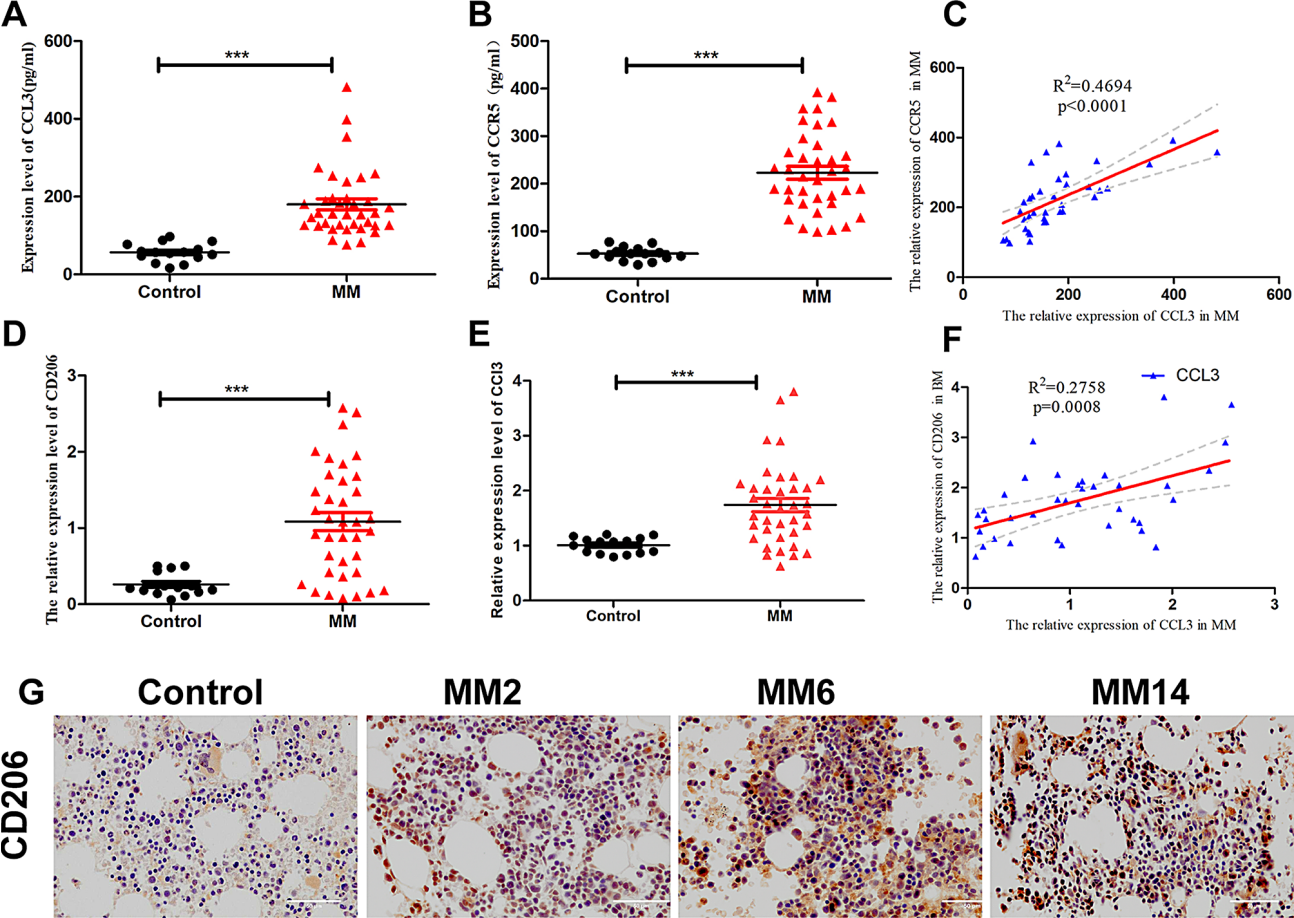
Expression of CCL3/CCR5 and the CD206 expression score of M2 macrophages in MM patients. **A**-**B**: CCL3 and CCR5 expression levels in BM were measured by ELISA (*n* = 37 MM patients and *n* = 15 healthy donors). **C**: Correlation between CCR5 and CCL3 expression levels. **D**: CD206 expression scores of M2 macrophages in BM tissues. **E**: mRNA expression levels of CCL3 in MM Bone Marrow (BM) samples measured by qRT-PCR. **F**: Correlation between CCL3 levels and the CD206 expression score. **G**: Representative images of CD206 + macrophage content in control and MM specimens by IHC staining (×200). (IHC, immunohistochemistry, *p < 0.05, **p < 0.01, ***p < 0.001)

To explore the potential role *of CCL3/CCR5 in* MM, we evaluated *expression* levels in the BM plasma of MM patients. The CCL3 and CCR5 levels were significantly higher in the MM patients (180 pg/ml ± 14.25,222.9 pg/ml ± 13.55) than in healthy individuals (56.81 pg/ml ± 6.048,52.71 pg/ml ± 3.616) (*p* < 0.001) (Fig. 1A-B). Moreover, the levels of CCL3 and CCR5 were positively associated with each other (R^2^ = 0.4694) (Fig. 1C).

To further investigate whether the elevated levels of CCL3 were associated increased numbers of M2 macrophages, we examined the expression of CCL3 mRNA and CD206 in BM neutrophils and paraffin-embedded BM tissue samples from patients with MM as compared to healthy controls. Both the relative expression of CCL3 mRNA and the CD206 expression scores of M2 macrophages were significantly higher in MM patients (1.741 ± 0.122, 1.084 ± 0.119) compared to healthy donors (1.01 ± 0.037, 0.262 ± 0.038) (Fig. 1D-E, G). Furthermore, elevated CCL3 expression was associated with CD206^+^ M2 macrophage subsets (R^2^ = 0.2758) (Fig. 1F). Collectively, these results suggest that CCL3/CCR5 and M2 macrophage polarization are characteristics of MM associated with advanced disease.

### MM cells promote in vitro macrophage M2 polarization that is mediated by autocrine CCL3

**Fig. 2:**
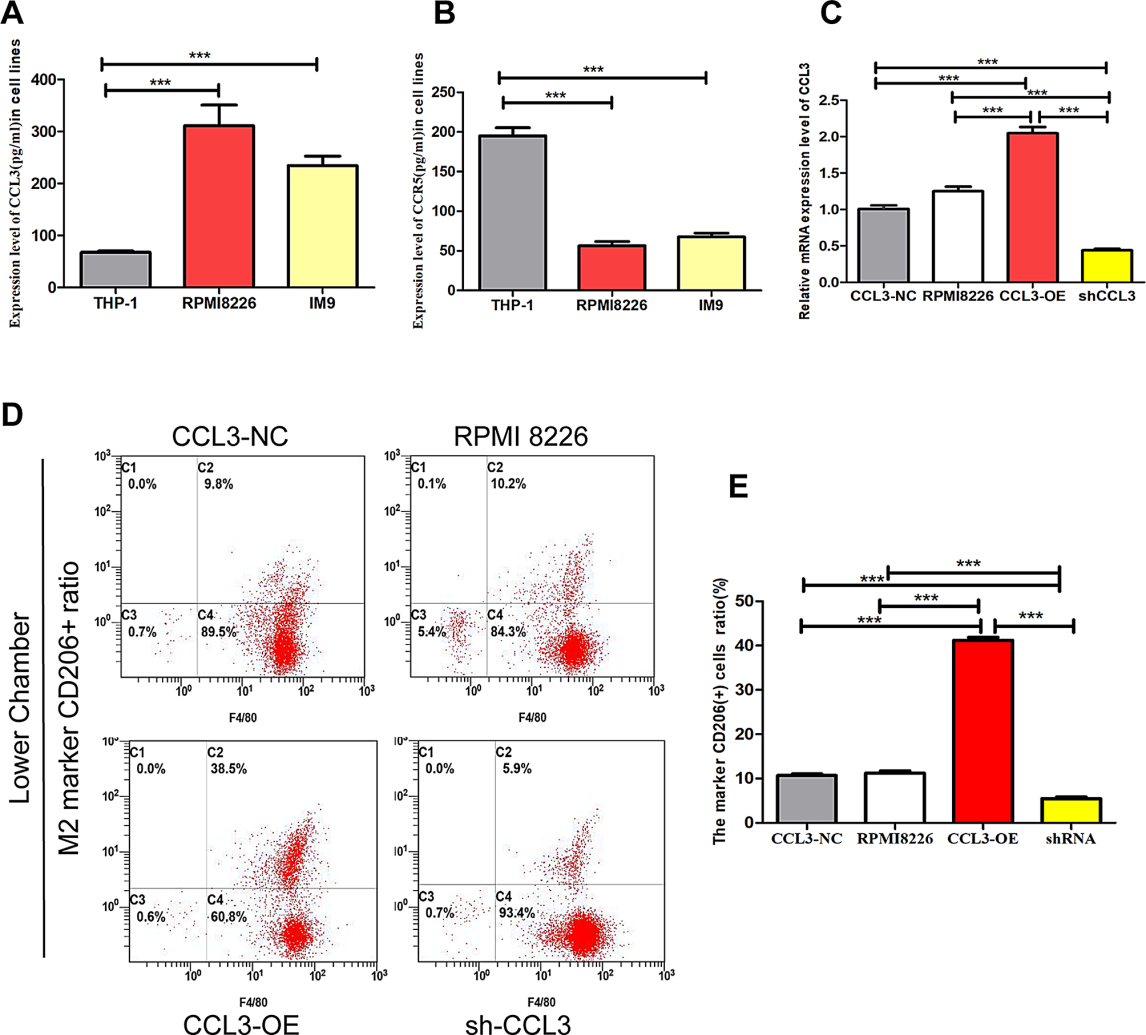
MM cells promote macrophages towards M2 polarization via autocrine CCL3. **A**-**B**: Expression levels of CCL3 and CCR5 mRNA in monocytic THP-1 and MM RPMI 8226 and MM IM9 cell lines was evaluated by qRT-PCR. **C**: Verification of overexpression (CCL3 OE) and knockdown (shCCL3) RPMI 8226 cells by qRT-PCR. **D**: Flow cytometric analysis of CD206 + cells after co-culture of THP-1 cells with CCL3-NC RPMI 8226 cells, RPMI 8226 cells, CCL3-OE RPMI 8226 cells or shCCL3 RPMI 8226 cells. **E**: Quantification of the differences in CD206 + cell levels among groups

To verify the autocrine or paracrine levels of CCL3 and CCR5 in BM MM cells, we examined CCL3 and CCR5 protein levels in culture supernatants. The levels of CCL3 were higher in human MM cell lines than in THP-1 human monocyte cells (Fig. 2A); however, THP-1 cells secreted higher levels of CCR5 (Fig. 2B). These results raise the possibility that CCL3-mediated macrophage recruitment occurs through autocrine CCL3 and paracrine CCR5 signaling.

To evaluate the role of CCL3 in M2 macrophage polarization, we established CCL3 knockdown and overexpression RPMI 8226 cells using shRNA and lentiviral constructs (Fig. 2C), which we co-cultured with PMA-stimulated THP-1 macrophages. Flow cytometry results revealed that the number of M2 macrophages was significantly increased in the CCL3-OE and decreased in the shRNA-CCL3 group as compared to the control group (Fig. 2D-E). Together, these results suggest that CCL3, which is highly expressed in MM cells, promotes M2 polarization of macrophages in the BM.

### *CCL3 promotes* macrophage M2 polarization *via the CCR5 receptor*

**Fig. 3:**
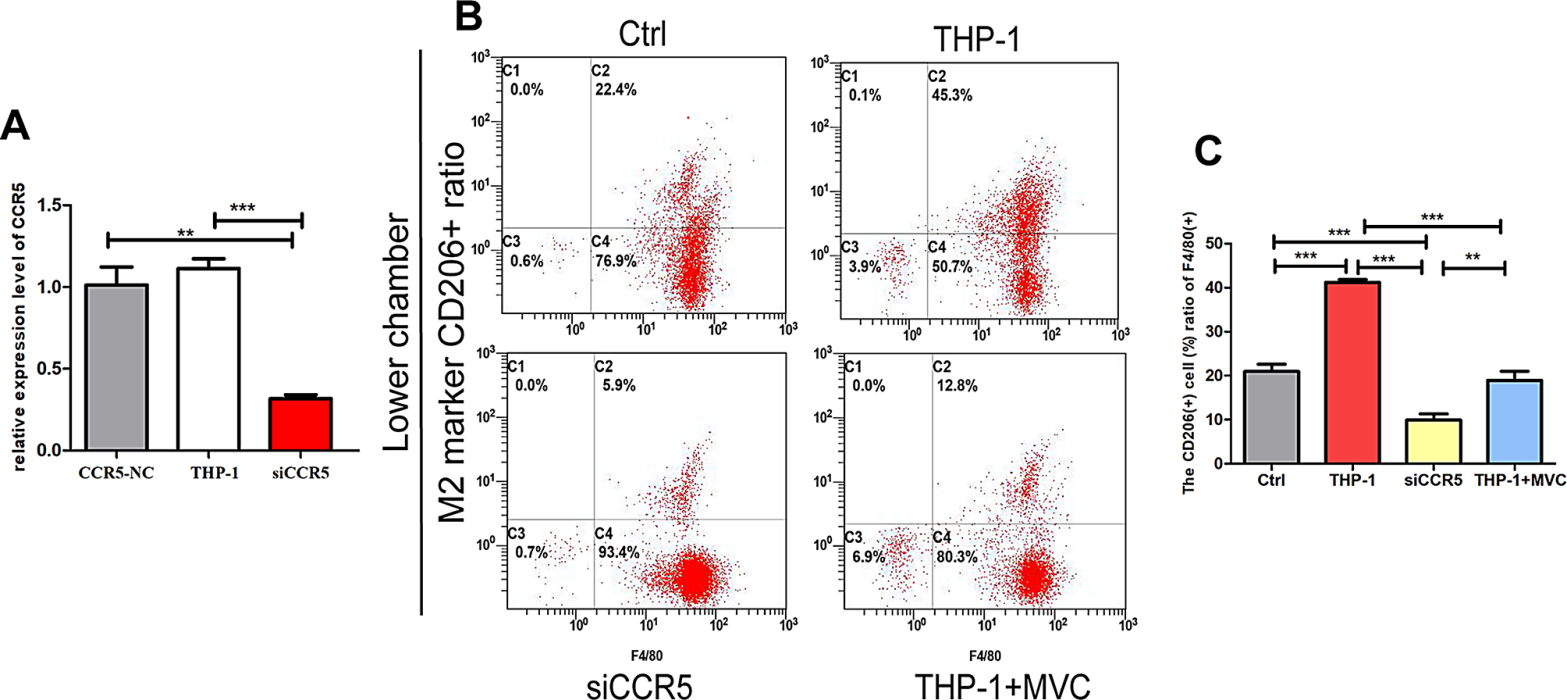
CCL3 promotes macrophages towards M2 polarization through the CCR5 receptor. **A**: Relative expression of CCR5 mRNA in transfected THP-1 cells. **B**: Flow cytometric analysis of CD206 + cells after co-culture of CCL3-OE RPMI 8226 cells with undifferentiated THP-1 cells (Ctrl), THP-1 cells differentiated with PMA, siCCR5 THP-1 cells differentiated with PMA or THP-1 cells treated with MVC after PMA induction. **C**: Quantification of differences in CD206 + cell levels among groups

To further investigate whether CCL3 mediates M2 macrophage polarization through CCR5, we used siRNA to knock down CCR5 expression in THP-1 cells (Fig. 3A). We then repeated co-culture assays with CCL3-OE RPMI 8226 cells and control or CCR5 knockdown THP-1 cells. The results suggest that CCR5 downregulation in THP-1 cells significantly attenuated the ability of CCL3-OE MM cells to promote macrophage polarization towards M2. For additional evidence, we used the CCR5 inhibitor MVC to block CCR5 in the co-culture assay. MVC treatment significantly weakened the ability of CCL3 to promote M2 polarization (Fig. 3B-C), thus further supporting a role for CCL3 in promoting macrophage polarization towards M2 via the CCR5 receptor.

### CCL3/CCR5-induced M2 macrophage polarization is mediated via activation of the PI3K/AKT/RhoA pathway

**Fig. 4:**
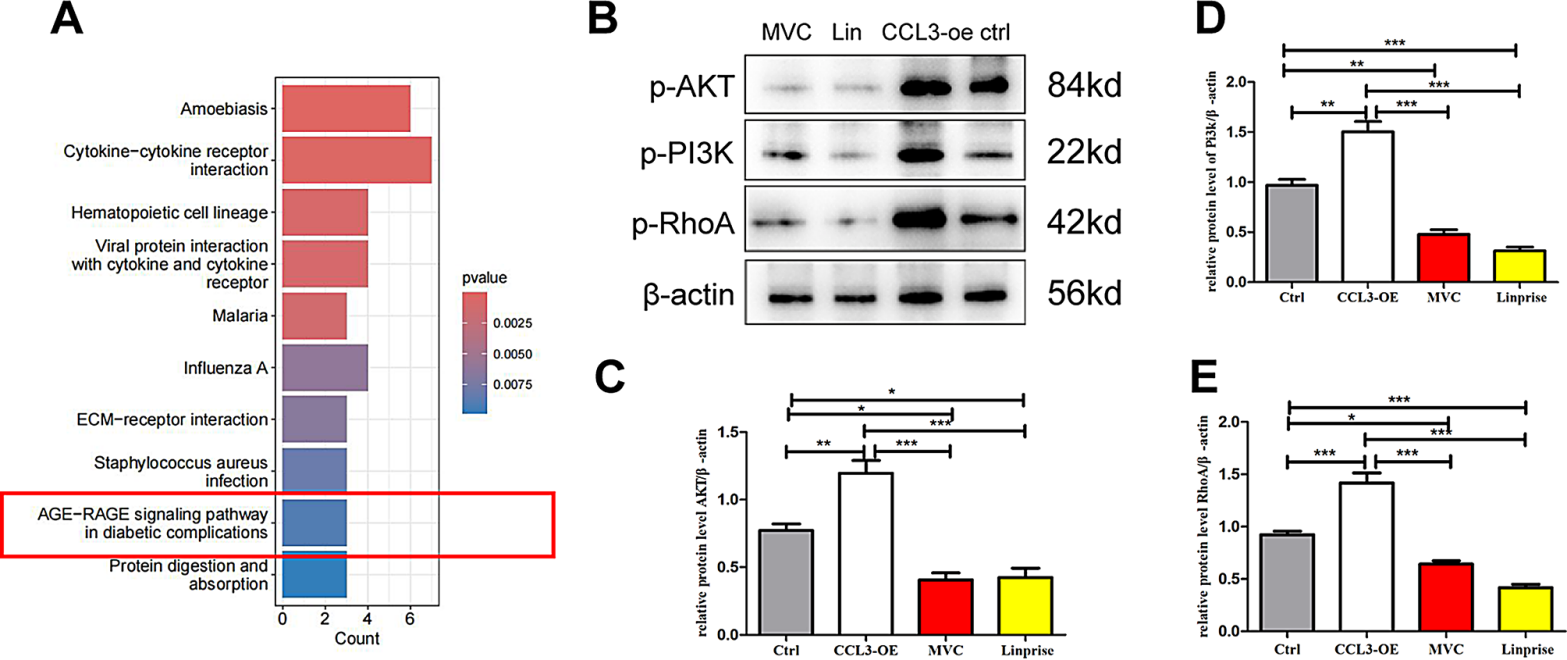
CCL3/CCR5-induced M2 macrophage polarization is mediated by PI3K/AKT/RhoA pathway activation. **A**: Differentially expressed genes identified by transcriptome sequencing were analyzed by KEGG enrichment analysis using the GSE215012 dataset. **B**: Western blot analysis of the protein levels of p-PI3K, p-AKT, and p-RhoA protein expression in M2-macrophages before and after treatment with 100 µM MVC (CCR5 inhibitor) or linperlisib (PI3K inhibitor) for 24 h. β-actin was used as a normalization control. **C**–**E**: The relative expression of p-PI3K, p-Akt, and p-RhoA protein in each group. (MVC; CCL3-NC co-cultured with THP-1-derived M2 macrophages + MVC; linperlisib, CCL3-NC co-cultured withTHP-1-derived M2 macrophages + linperlisib; CCL3-OE, CCL3-OE co-cultured with THP-1-derived M2 macrophages; Ctrl, CCL3-NC co-cultured with THP-1-derived M2 macrophagesl)(p-PI3K, phosphorylated PI3K; p-AKT, phosphorylated AKT; p-RhoA, phosphorylated RhoA; MVC, maraviroc; Lin, linperlisib)

To further ascertain which pathways are activated by CCL3 and CCR5 in promoting M2 macrophage polarization, we performed KEGG enrichment analysis of transcriptomic sequencing data. The AGE-RAGE signaling pathway was shown to be activated (Fig. 4A). Moreover, PI3K/AKT/mTOR signaling was predicted to be activated within this pathway, with RhoA—a downstream target gene of AKT—playing a role in modifying macrophage polarity (Figures S1-S2). Our group previously showed that M2 macrophage polarization is activated PI3K/AKT signaling in MM cells [18]. Therefore, we hypothesized that CCL3-mediated M2 polarization may be mediated via the PI3K/AKT/RhoA signaling pathway, which in turn may promote MM progression.

To evaluate our hypothesis, we analyzed the expression levels of PI3K/AKT/RhoA in macrophages using our co-culture system. The results demonstrated that the phosphorylated levels of PI3K (p-PI3K), AKT (p-AKT), and RhoA (p-RhoA) were higher in THP-1-derived M2 macrophages that were co-cultured with CCL3-OE cells as compared with CCL3-NC cells. As expected, the levels were reduced when the THP-1-derived M2 macrophages were treated with the PI3K inhibitor Linperlisib, while macrophages treated with the CCR5 inhibitor MVC had the lowest level of AKT/PI3K/Rho activation (Fig. 4B-E). These findings confirm that CCL3/CCR5 promotes M2 macrophage polarization by activating the PI3K/AKT/RhoA pathway in mononuclear-macrophages cells within the myeloma BMME.

### M2 *macrophages* support MM cell survival and proliferation *and* protect MM cells from *Bortezomib*-induced apoptosis

**Fig. 5:**
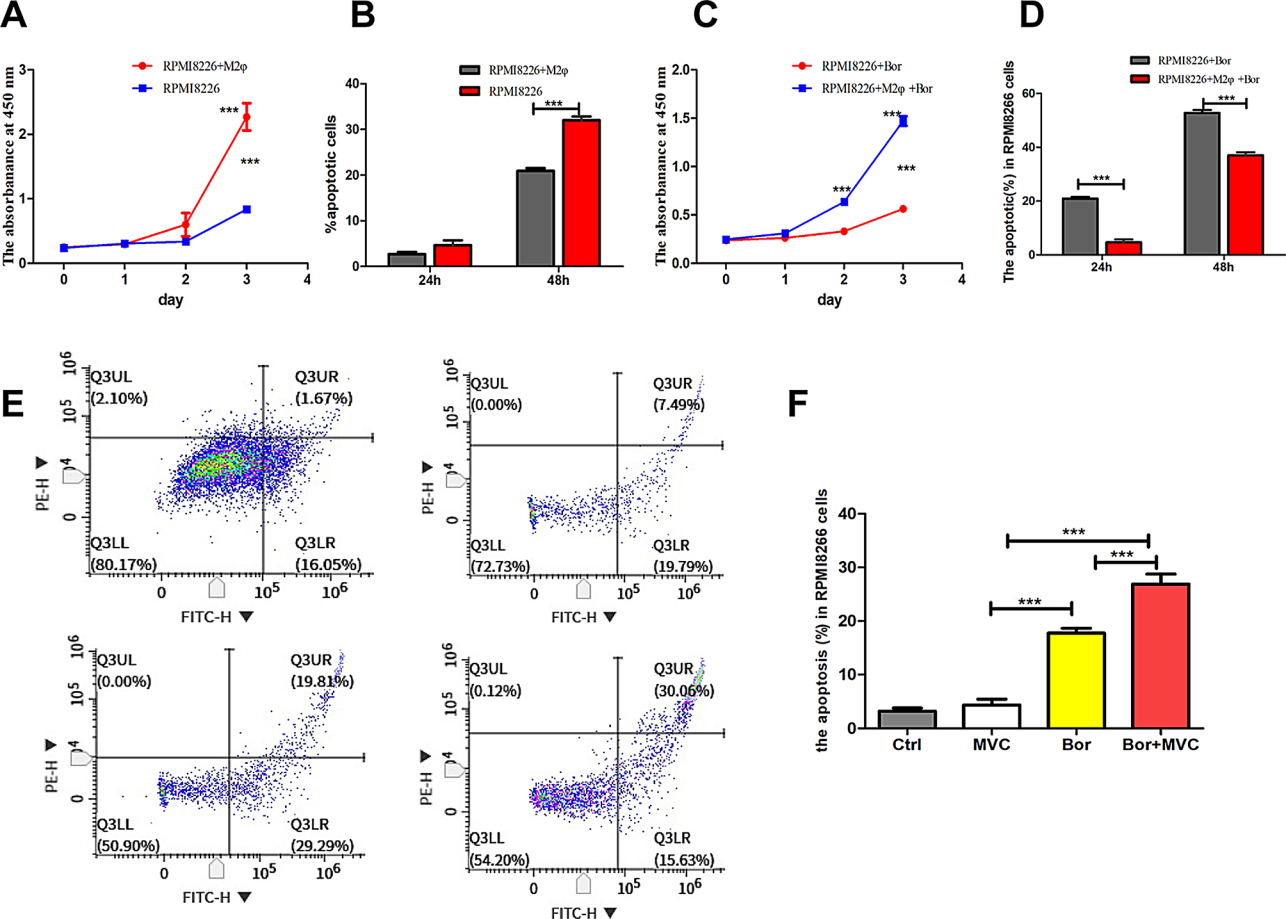
M2 Macrophages support MM cell survival and proliferation and protect MM cells from Bortezomib-induced apoptosis. **A**-**B**: THP-1-derived M2 macrophages promote cell proliferation and inhibit cell apoptosis and C-**D**: protect MM cells from Bortezomib-induced apoptosis of RPMI 8226 cells. **E**–**F**: RPMI 8226 cells co-cultured with THP-1 cells treated with MVC, Bor or Bor combined with MVC for 24 h. The percentages of cells in the fourth quadrant were calculated, and the results were plotted as histograms. Results are present as mean ± S.D. (*n* = 4), Statistical analysis was carried out with the one-way ANOVA

The infiltration of TAMs into the BMME is a critical driver of tumor progression [21]. Therefore, we sought to evaluate the impact of myeloma-macrophage interactions on the viability of MM cells. Our results demonstrate that THP-1-derived M2-macrophages significantly increased the proliferation and inhibited the apoptosis of RPMI 8226 cells (Fig. 5A-B). Moreover, incubation with M2 macrophages significantly protected RPMI 8226 cells from Bortezomib-induced cell apoptosis (Fig. 5C-D). For additional evidence of the role of CCR5 in M2-mediated MM cell protection, we evaluated RPMI 8226 cell apoptosis after incubation with M2 macrophages treated Bortezomib in the presence or absence of MVC. The results demonstrated that MVC significantly enhanced Bortezomib-induced apoptosis of RPMI 8226 cells (Fig. 5E-F). These results indicate that CCR5 blockade enhances the M2 macrophage-induced drug-sensitivity of MM cells to Bortezomib, indicating its potential as a therapeutic strategy.

*3.6 The combination of* CCL3–CCR5 *blockade and* Bortezomib *prevents* M2 *macrophage* polarization and *MM* progression in *a mouse model*.

**Fig. 6:**
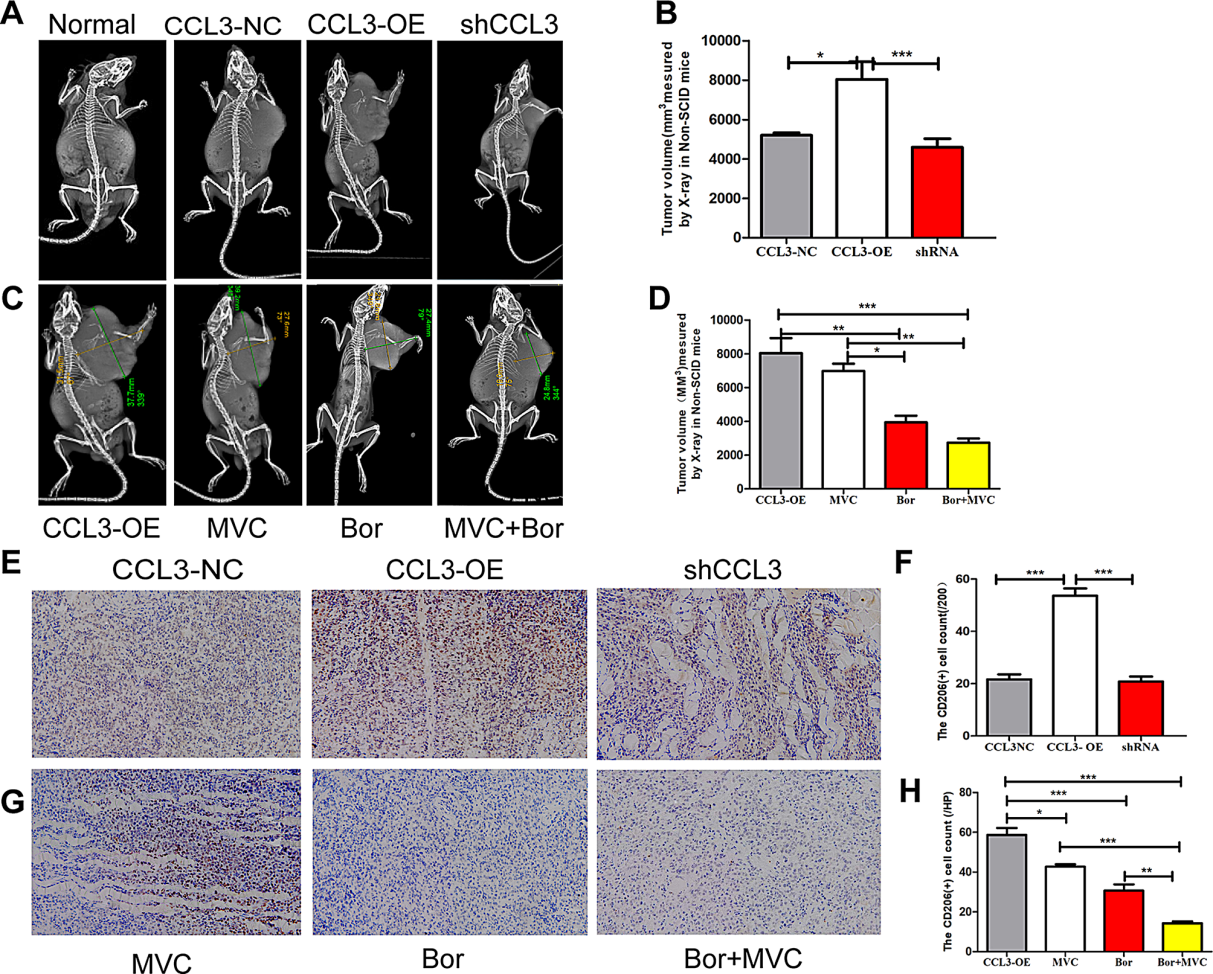
CCL3-CCR5 axis blockade prevents M2 macrophage polarization and MM progression in vivo. **A**: Tumor volumes in mice injected with no cells (Normal), CCL3-NC RPMI 8226 cells, CCL3-OE RPMI 8226 cells, or shCCL3 cells. **B**: Quantification of the results from panel **A**. **C**: Tumor volumes in mice injected with CCL3-OE cells and then treated with MVC, Bor or Bor combined with MVC 24 h. **D**: Quantification of the results from panel **C**. **E**-**H**: Representative images and quantification of the CD206 + macrophage content in xenograft NOD/SCID mouse model tumor tissues by the IHC staining (×40)

To evaluate the in vivo role of the CCL3/CCR5 axis in the progression of MM, we established a mouse xenograft model in which NOD/SCID mice were subcutaneously transplanted with CCL3-OE, CCL3-NC or sh-CCL3 RPMI 8226 cells. The tumor volume was significantly larger in the CCL3-oe group than in the shCCL3 or CCL3-NC group (Fig. 6A-B). Furthermore, the tumor volume was not significantly reduced by MVC treatment; however, Bortezomib reduced the tumor volume, though the combination of MVC and Bortezomib treatment was most effective in reducing the tumor volume (Fig. 6C-D). IHC staining further confirmed that the percentage of CD206-positive macrophages was upregulated in the CCL3-OE group compared to the RPMI 8226 CCL3-sh and CCL3-NC group (Fig. 6E-F) and downregulated by MVC, Bortezomib, and most dramatically, by the two drugs in combination (Fig. 6G-H). These results indicate that MM cells recruit macrophage infiltration toward M2 polarization by secreting CCL3, and that the CCR5 inhibitor MVC enhances the sensitivity of Bortezomib treatment.

## Discussion

In MM development, the interplay between myeloma and immune cells in the BMME plays a pivotal role [6]. Studies indicate that TAMs, primarily including M2 macrophages, contribute significantly to drug resistance and disease progression [22]. The recruitment of macrophages and polarization of TAMs are orchestrated by cytokines and chemokines derived from tumor cells and immune component cells in the BMME. Among these interactions, chemokines play a crucial role in directing the behavior of macrophages [23, 24].

M2 macrophages are characterized by their secretion of IL-10, PGE2, and TGF-β via the arginase pathway, promoting Th2 immune response and facilitating tumor progression and invasion in MM by inducing angiogenesis and suppressing the host’s immune response [25]. Furthermore, CCL3, secreted by monocytes and macrophages, is implicated in TAMs promotion of tumor cell proliferation and development [26]. In pediatric high-grade gliomas, CCL3 facilitates M2 macrophage infiltration into the tumor microenvironment [27]. Consequently, knocking out the CCL3 gene in mouse models inhibits M2 macrophage infiltration and prolongs survival [28]. Moreover, the binding of CCL3 to CCR5 has been demonstrated to enhance M2 macrophage polarization [29]. We demonstrated that in the MM BMME, elevated levels of CCL3, CCR5, and M2 macrophages are positively correlated, suggesting that the CCL3-CCR5 interaction may promote M2 macrophage infiltration. Notably, CD206 is a commonly used marker for identifying M2 macrophages for its high expression on M2 macrophages, but there are limitations of using CD206 as the sole marker for M2 polarization. Further research should use a panel of markers or functional assays to more accurately characterize macrophage polarization states and strengthen these results.

CCR5, a seven-transmembrane G protein-coupled receptor, is expressed on a variety of cell types, such as T cells, macrophages, and various tumor cells [30]. It has been reported that CCR5 expression is increased in the myeloma microenvironment, leading to leukocyte chemotaxis, immunosuppression, and tumor cell migration and infiltration, ultimately promoting tumor proliferation and spread. Similarly, in solid tumors such as breast cancer and thyroid cancer, CCL3 binding to CCR5 upregulates MMPs and activates the NF-κB pathway, thereby enhancing cancer cell invasiveness and migration [31, 32]. In this study, we employed a co-culture system to demonstrate that CCL3 promotes the polarization of THP-1 cells to M2 macrophages in MM. Overexpression of CCL3 increased the number of M2 macrophages, while CCR5 inhibitors reduced M2 polarization, suggesting that blockade of the CCL3-CCR5 pathway inhibits macrophage polarization to M2. Additionally, we demonstrated that M2 macrophages promote tumor cell proliferation, inhibit apoptosis, and decrease sensitivity to Bortezomib, indicating a role in drug resistance. Further validation in bortezomib-resistant cell lines would strengthen these results. Furthermore, carfilzomib, a proteasome inhibitor as well as bortezomib used in the treatment of multiple myeloma, was showed to effectively rewire tumor microenvironment through reprogramming TAMs into M1-like macrophages [33]. Experiments need to be conducted to compare the differences and similarities in mechanisms of these two proteasome inhibitors.

We demonstrated that the molecular mechanisms underlying M2 polarization involve PI3K, AKT, and RhoA activation, suggesting a pathway by which tumor cells induce M2 macrophage infiltration. Inhibition of the activation of each of these signaling molecules in response to CCR5 antagonist and PI3K antagonist administration further supports the involvement of the PI3K/AKT/RhoA pathway in the polarization of macrophages. CCR5 may activate PI3K/AKT/RhoA pathway via CCR5-coupled G proteins.

Maraviroc, a competitive antagonist of the CCR5 receptor, was originally designed as an entry inhibitor for HIV infections. Owing to mounting importance of the CCR5 axis in cancer, maraviroc turned out to be an immediately available drug for therapeutic purposes. Although the CCR5 antagonist MVC alone did not significantly reduce tumor volume, combined treatment with Bortezomib led to significant tumor shrinkage, supported by a synergistic effect between CCR5 antagonists and chemotherapy in inhibiting tumor proliferation and promoting apoptosis. This suggests that targeted inhibition of macrophage polarization may complement other therapeutic strategies, such as chemotherapy or immunotherapy, offering a promising treatment approach for MM. MVC was the first Food and Drug Administration-approved drug for treating HIV infection and is now being repurposed for cancer therapy [34]. Therefore, further research is warranted to validate the use of MVC for MM treatment, including the potential toxicity concerns, dose limitations of MVC in MM patiesive analysis of the immune microenvironment and studies to extend these findings to the clinic.

## Conclusion

In summary, we characterized how CCL3 secreted by MM cells in an autocrine manner can drive M2 polarization and recruit CCR5 to the tumor area through paracrine signaling, thereby promoting myeloma progression and inducing drug resistance. Targeting of the CCL3/CCR5 axis with MVC in combination with Bortezomib offers a promising therapeutic strategy against myeloma, as demonstrated by both in vitro studies and xenograft mouse models.

## Methods

### Selection of MM patient and healthy donors

Between October 2019 and April 2022, BM samples were collected at the Affiliated Hospital of Guilin Medical University from 37 MM patients and 15 healthy donors. The diagnosis and disease assessments were performed according to the International Myeloma Working Group (IMWG) International Staging System for MM [20]. The inclusion criteria for the experimental group were: (1) diagnosis of MM according to the IMWG criteria and evidence of relapsed/ refractory or remission MM; and (2) age between 18 and 80 years. The exclusion criteria were: (1) secondary MM (patients with a prior diagnosis of another malignancy) or mixed MM (diagnoses of both MM and another malignancy); (2) autoimmune diseases or other neoplastic diseases affecting the rheumatic system; (3) concurrent hematopoietic or lymphoid tissue disorder or history of a solid tumor; and (4) pregnancy or lactation. Approval for the study was obtained from the Ethics Committee of the Affiliated Hospital of Guilin Medical University (No.20210027). The study was conducted in accordance with the tenets of the Declaration of Helsinki.

### Specimen processing and handling

BM aspirates and tissues were obtained from the study participants by a skilled clinician using standardized BM aspiration techniques. Blood samples were immediately collected into EDTA anticoagulation tubes, and BM tissues were promptly fixed in formalin. Within 30 min after collection, the blood samples were centrifuged at 1500 g for 20 min at 4 °C. Subsequently, the plasma was carefully aspirated and stored in Eppendorf tubes at 80 °C until use in further analyses by enzyme-linked immunosorbent assay (ELISA) or other relevant procedures.

In parallel, BM was separated according to the standard Ficoll-Paque method (Solarbio, Beijing, China), and mononuclear cells were harvested and cryopreserved in liquid nitrogen. The mononuclear cells were stored at 80 °C for subsequent real-time quantitative reverse transcription PCR (qRT-PCR) assay. Additionally, samples of the BM tissues were embedded in paraffin for use in immunohistochemical (IHC) analyses.

### Cell lines, cell culture, and lentiviral transduction

The human MM RPMI 8226, IM9 cell lines and human monocyte THP-1 cell lines were purchased from the Hematology Research Center of China Medical University. The cells were cultured in RPMI 1640 (Gibco, Grand Island, NY, USA) supplemented with 10% fetal bovine serum (FBS; Solarbio, Beijing, China), 50 µg/mL penicillin and 50 µg/mL streptomycin (Solarbio, Beijing, China), at 37 °C in a humidified 5% CO_2_ incubator. The siRNAs for human CCR5 were purchased from GENE (Shanghai, China). THP-1 cells were transiently transfected with siRNA utilizing Lipofectamine 3000 Transfection Reagent (L3000-015, Invitrogen, Carlsbad, CA, USA), according to the protocol provided by the manufacturer. Lentivirus vectors carrying shRNA-CCL3 or CCL3 OE plasmids were constructed, and RPMI 8226 cells were infected with corresponding lentiviruses to downregulate or upregulate the expression of CCL3 in MM cell lines. Bortezomib (MedChemExpress, Monmouth Junction, NJ, USA) for anti-MM cells, Maraviroc (MVC, Targetmol, Boston, MA, USA) for blocking CCR5 chemoresistance and Linperlisib (Hengrui, Lianyungang, China) for blocking PI3K/AKT were directly added in the culture medium at 100 nM for 48 h.

### MM cell xenograft NOD/SCID mouse model

NOD/SCID mice (male, 4–6 weeks old) were procured from Hunan Slaughter Kingda Animal Laboratory (China). All mice were housed in individually ventilated cages under specific pathogen-free conditions with controlled lighting, humidity (40-70%), and temperature (15–25 °C) in the animal facility at the Guilin Medical University. The Animal Ethics Committee of Guilin Medical University (NO. GLMC202003073) approved the animal experiments, which adhered to the 8th edition of the Guidelines for the Care and Use of Experimental Animals. The mice were subcutaneously injected in the forelimb with RPMI 8226, RPMI 8226/shCCL3 or RPMI 8226/CCL3-OE cells (5 × 10^6^ cells per site). For injection, the cells were suspended in 50 µL of FBS and combined with an equal volume of Matrigel (Corning, Corning, NY, USA). The tumor size was measured at designated intervals, with volumes calculated using the formula V = 4π/3 × (L/2) × (S/2)^2^, where L represents the largest diameter and S the perpendicular diameter. For in vivo drug therapy, the mice were randomly assigned to different groups, ensuring similar average tumor volumes of approximately 2–3 mm prior to drug treatment. Treatments included Bortezomib (0.5 mg/kg, twice a week for 1 week, Bor), Maraviroc (100 mg/kg, MVC) intraperitoneally for 4 consecutive days, or vehicle (Ctrl), administered by intraperitoneal injection every 7 days for the specified duration. All mice were euthanized after an additional 2 weeks. Before the mice were sacrificed, a molybdenum target X-ray (PerkinElmer, Waltham, MA, USA) was used to image the tumor volume in mice. Following euthanasia, tumors were excised, fixed in 10% formalin, embedded in paraffin, and sectioned at 3 μm thickness for subsequent IHC staining.

### Immunohistochemistry (IHC)

IHC was performed using paraffin-embedded sections of BM biopsy tissues according to the standard Envision protocol (Dako, Glostrup, Denmark). Primary antibodies against *α*-SMA and CD206 (Biolegend, San Diego, CA, USA) were used for IHC. Briefly, tissue sections (approximately 3 μm in thickness) were mounted on slides, deparaffinized in xylene, and rehydrated through a graded series of alcohol solutions. The slides were then subjected to antigen retrieval in 10 mM sodium citrate buffer (pH 8.0) at 100 °C for 10 min. The staining intensity was independently evaluated and scored by three experienced pathologists. For quantification of CD206 levels, five representative fields per slide were examined under a light microscope (Olympus BX51, Tokyo, Japan). The percent positivity of CD206 staining was evaluated on a scale from 0 to 4, where 0 indicated less than 5%, 1 represented 5–25%, 2 corresponded to 26–50%, 3 denoted 51–75%, and 4 indicated greater than 75%. The staining intensity was also assessed using a 4-point scale: 0 (no staining), 1 (weak staining, light yellow), 2 (moderate staining, yellowish brown), and 3 (strong staining, brown). The CD206 expression score was then calculated by multiplying the percent positivity score by the staining intensity score. CD206 expression levels were categorized based on the resulting scores as low (0–4), medium (5–8), or high (9–12).

### Co-culture of THP-1 and RPMI 8226 cells for assessment of M2 macrophage polarization

THP-1 cells, with or without siRNAs, were exposed to conditioned medium containing Phorbol 12-myristate 13-acetate (PMA) (Sigma-Aldrich, St. Louis, MO, USA) to induce macrophage differentiation, followed by co-culture with RPMI 8226 cells. Specifically, THP-1 cells (1 × 10^6^ cells per well) were seeded in the lower chamber of a 6-well transwell apparatus with a 0.4-µm pore size membrane and stimulated with 150 nM PMA and 20 ng/ml interleukin-4 (IL-4). After 24 h, the differentiated THP-1 cells were thoroughly washed with PBS to remove residual PMA. RPMI 8226 cells (5 × 10^5^ cells per well), transfected with either shRNA-CCL3 or CCL3-OE, were then placed in the upper chamber and co-cultured with the PMA-induced THP-1 cells or siCCR5 without direct contact. After 48 h, the polarized THP-1 macrophages were collected for subsequent assays. Where indicated, the macrophages were pre-cultured with a CCR5 inhibitor MVC or PI3K inhibitor (Linprise), which were added to the lower chambers for 48 h.

### Cell counting Kit-8 (CCK8) assay

Cell viability and proliferation was assessed using Cell Counting Kit-8 Reagent (CCK-8, MedChemExpress, Monmouth Junction, NJ, USA). In brief, RPMI 8226 and transfected cells were plated into 96-well plates and cultured for 24, 48–72 h. Then 10 µL CCK8 solution was added per well, and the cultures were incubated for another 4 h. Finally, the absorbance at 450 nm was measured using a spectrophotometric plate reader for determination of cell proliferation.

### Enzyme-linked immunosorbent assay (ELISA)

CCL3 and CCR5 supernatant levels in the BM samples from MM patients, THP-1 cells, and human MM cells were measured by ELISA using commercially available kits (QIAGEN, Hilden, Germany), as described by the manufacturer. The standard curve was based on the measured OD values of the standard, and the results were expressed in pg/ml.

### RNA extraction and quantitative real-time polymerase chain reaction (qRT‑PCR)

To assess the mRNA expression levels of PI3K, AKT, and RhoA in macrophages, the following protocol was implemented: (1) total RNA was extracted from macrophages using Trizol reagent (TaKaRa Bio, Shiga, Japan); (2) RNA was then reverse transcribed into cDNA using a cDNA First Strand Synthesis Kit (MonScript™ RTIII All-in-One Mix with dsDNase REF: MR05101) (Mona Bio, Beijing, China) according to the manufacturer’s directions; (3) PCR amplification was carried out using SYBR (MonAmp™ SYBR^®^ Green qPCR Mix [Low ROX]) (Mona Bio, Beijing, China) and an ABI 7500 system under the following conditions: a 20-µL total reaction volume, an initial pre-denaturation step at 95 °C for 10 min, followed by denaturation at 95 °C for 10 s, annealing at 60 °C for 10 s, and extension at 72 °C for 30 s, for a total of 40 cycles. β-actin was employed as the internal control for normalization, and the relative mRNA expression levels were quantified using the 2^−ΔΔCt^ method. Each sample was tested in triplicate, and the mean values were calculated. All primers (Supplementary Table 1) were synthesized by Sangon Biotech.

### Flow cytometric analysis

To evaluate the expression of the M2 macrophage marker CD206, as well as the cell apoptosis rate, macrophages were collected and washed with PBS, and the cell viability was confirmed to be 80–90%. After counting, the cells were centrifuged at 1200 rpm for 5 min and resuspended in FACS buffer (2 mM EDTA, 0.1% BSA) at a concentration of 2 × 10^6^ cells/mL. The cells were incubated in the dark at 4 °C with blocking antibody (20 µg/mL) on a bidirectional shaker for 30 min, followed by an additional 50 min with fluorophore-conjugated primary or isotype control antibody. Subsequently, the cells were stained with anti-F4/80-FITC, anti-CD11c-PE, and anti-CD206-APC (MedChemExpress, Monmouth Junction, NJ, USA) according to the manufacturer’s instructions. Finally, the cells were resuspended in 100 µL PBS, and the resulting cell suspension was transferred to a flow cytometry tube for analysis. The fluorescence intensity of fluorescein isothiocyanate was quantified using Flow-jo software (BD Biosciences, San Diego, CA, USA). Results were analyzed using FlowJo software version 10 (Treestar, Ashland, OR, USA).

### Western blotting

After whole cell lysis, protein concentrations were quantified according to manufacturer instructions (Beyotime; Shanghai, China). Equal amounts of protein samples were separated on a 10% sodium dodecyl sulfate-polyacrylamide gel electrophoresis gel and then transferred onto polyvinylidene difluoride membranes. The membranes were blocked at room temperature for 1 h with 5% skimmed milk in Tris-buffered saline with Tween 20. After blocking, the membranes were incubated overnight with primary antibodies specific to the target proteins, followed by a series of washes. The membranes were then incubated with secondary antibody, followed by further washing, developing, and film processing. Band intensities were quantified using ImageJ software 1.4.3.67 (National Institutes of Health, Bethesda, MD, USA), with β-actin serving as an internal loading control. A detailed summary of the antibodies used is presented in Supplementary Table 2.

### Bioinformatics Analysis

The macrophage polarization dataset, designated as GSE215012, was obtained from the Gene Expression Omnibus database. Differentially expressed genes were identified from the normalized dataset using the limma package in R, with the following criteria: |log2-fold change| ≥ 0.5 and adjusted P-value (adj. P) < 0.05. To identify biological pathways associated with macrophage M2 polarization, the differentially expressed genes were analyzed for pathway enrichment using the Kyoto Encyclopedia of Genes and Genomes (KEGG) database, with the “clusterProfiler” and “org.Hs.eg.db” packages in R. Using the ScanSite online tool (http://scansite.mit.edu), potential interactions among the AKT, PI3K, and RhoA domains were predicted based on protein sequence information and domain recognition patterns. This approach allows for the identification of functionally significant protein interaction sites, providing insights into key nodes and regulatory mechanisms within signaling networks.

### Statistical analysis

GraphPad Prism 9 (GraphPad Software, La Jolla, CA, USA) was utilized for graphical representation and statistical analysis of the data. Continuous variables were expressed as mean ± standard deviation (SD) from at least three independent experiments. Counts of M2 macrophages were reported as medians with ranges, while categorical data were presented as numbers (percentages). Before conducting comparative analyses, the normality of the data was evaluated using the Shapiro-Wilk test. For variables that followed a normal distribution, an unpaired two-tailed Student’s t-test was employed to compare differences between two groups. For data that did not follow a normal distribution, the Mann-Whitney U test was utilized. For data exhibiting homogeneity of variance, a one-way analysis of variance (ANOVA) was applied, while Welch’s ANOVA was employed for datasets where variance was not homogeneous. Correlations among different groups were determined using Spearman’s correlation coefficient. All statistical tests were conducted at a significance level of *p* < 0.05, with annotations for non-significance (ns), significance (*, *p* < 0.05; **, *p* < 0.01; ***, *p* < 0.001).

## Electronic supplementary material

Below is the link to the electronic supplementary material.

Supplementary Material 1

Supplementary Material 2: Supplementary Figure 1 Predicted Interactions of PI3K (p85 SH2 Domain) with AKT1 and RhoA Domains via ScanSite.

Supplementary Material 3: Supplementary Figure 2 Predicted Interactions of PI3K (p85 SH2 Domain) with AKT1 and RhoA Domains via ScanSite.

Supplementary Material 4: Supplementary Table1: Sequences of all primers.

Supplementary Material 5: Supplementary Table2: Information of antibody characteristics.

## Data Availability

The macrophage polarization dataset, designated as GSE215012, was obtained from the Gene Expression Omnibus database.
