# Supplementary figures and images for: Maraviroc enhances Bortezomib sensitivity in multiple myeloma by inhibiting M2 macrophage polarization via PI3K/AKT/RhoA signaling pathway in macrophages
Source: Cell Div. 2025 Feb 14;20:5. doi: 10.1186/s13008-025-00145-1 (PMC11829472; doi:10.1186/s13008-025-00145-1)

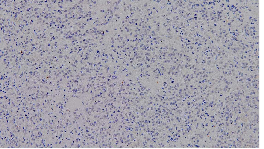

Supplement: Supplementary file 1 — Supplementary Material 1 [file 13008_2025_145_MOESM1_ESM.zip › ╘¡═╝/Bor MVC.tif]

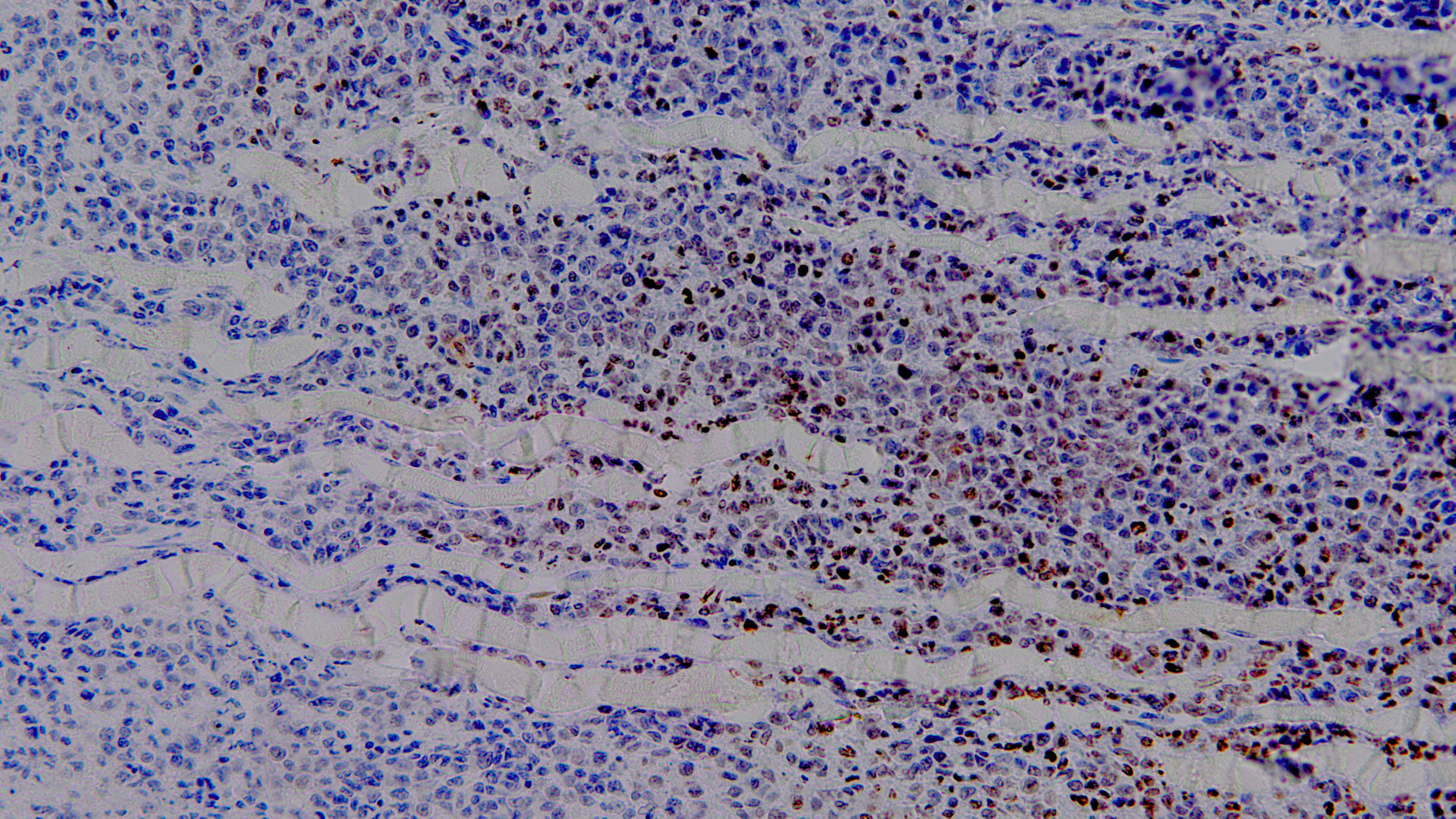

Supplement: Supplementary file 1 — Supplementary Material 1 [file 13008_2025_145_MOESM1_ESM.zip › ╘¡═╝/Bor.tif]

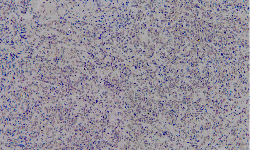

Supplement: Supplementary file 1 — Supplementary Material 1 [file 13008_2025_145_MOESM1_ESM.zip › ╘¡═╝/CCL3NC.tif]

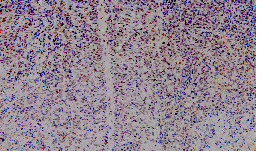

Supplement: Supplementary file 1 — Supplementary Material 1 [file 13008_2025_145_MOESM1_ESM.zip › ╘¡═╝/CCL3OE.tif]

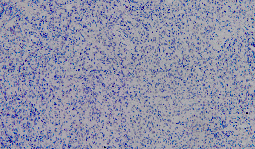

Supplement: Supplementary file 1 — Supplementary Material 1 [file 13008_2025_145_MOESM1_ESM.zip › ╘¡═╝/MVC.tif]

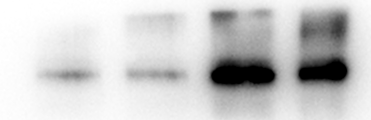

Supplement: Supplementary file 1 — Supplementary Material 1 [file 13008_2025_145_MOESM1_ESM.zip › ╘¡═╝/p-akt-11s.tif]

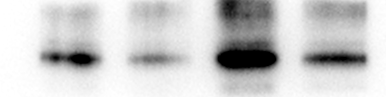

Supplement: Supplementary file 1 — Supplementary Material 1 [file 13008_2025_145_MOESM1_ESM.zip › ╘¡═╝/p-p13k-15s.tif]

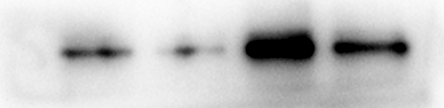

Supplement: Supplementary file 1 — Supplementary Material 1 [file 13008_2025_145_MOESM1_ESM.zip › ╘¡═╝/p-rhoa (2).tif]

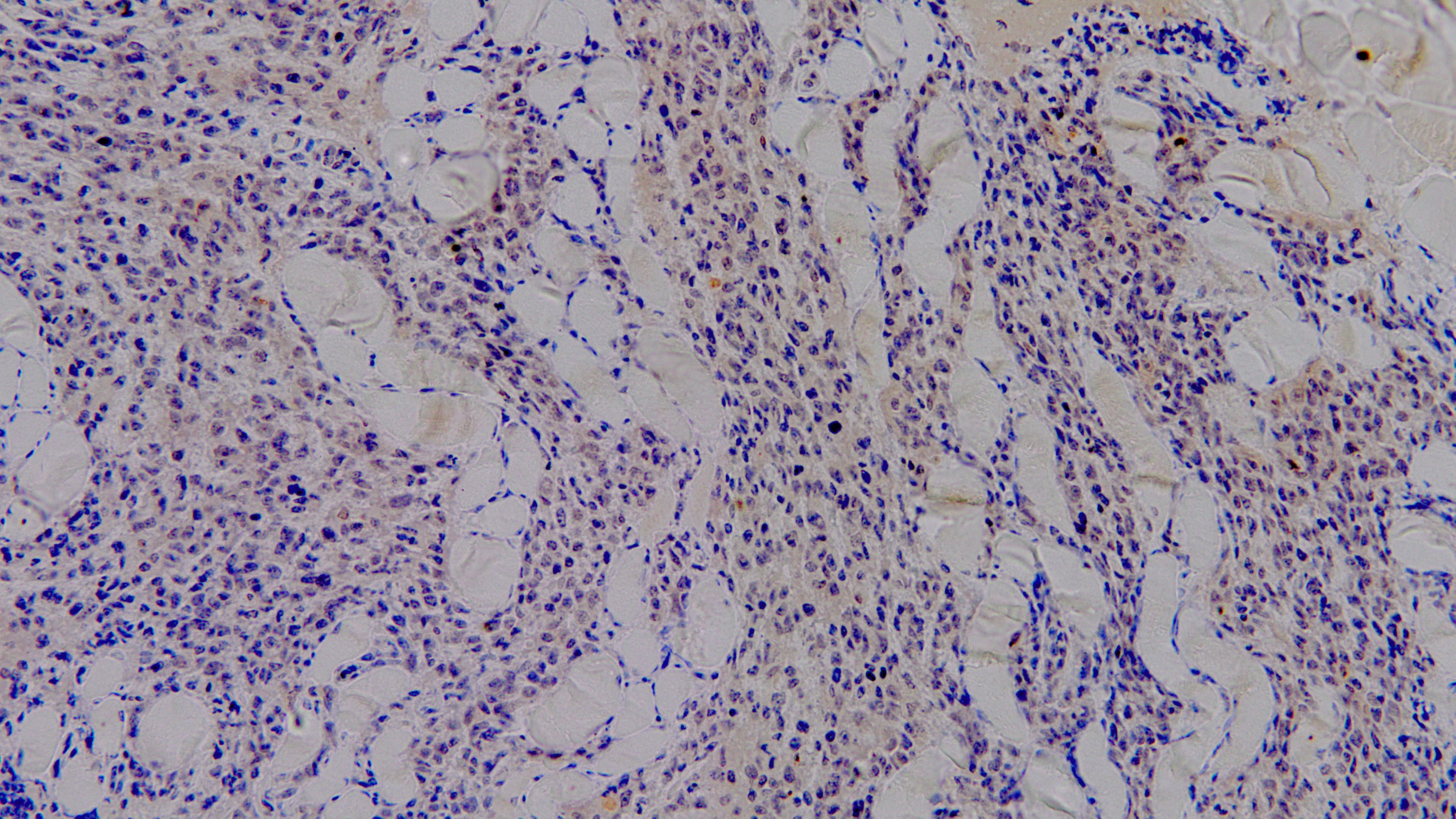

Supplement: Supplementary file 1 — Supplementary Material 1 [file 13008_2025_145_MOESM1_ESM.zip › ╘¡═╝/shCCL3.tif]

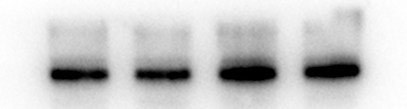

Supplement: Supplementary file 1 — Supplementary Material 1 [file 13008_2025_145_MOESM1_ESM.zip › ╘¡═╝/─┌▓╬2-26s.tif]

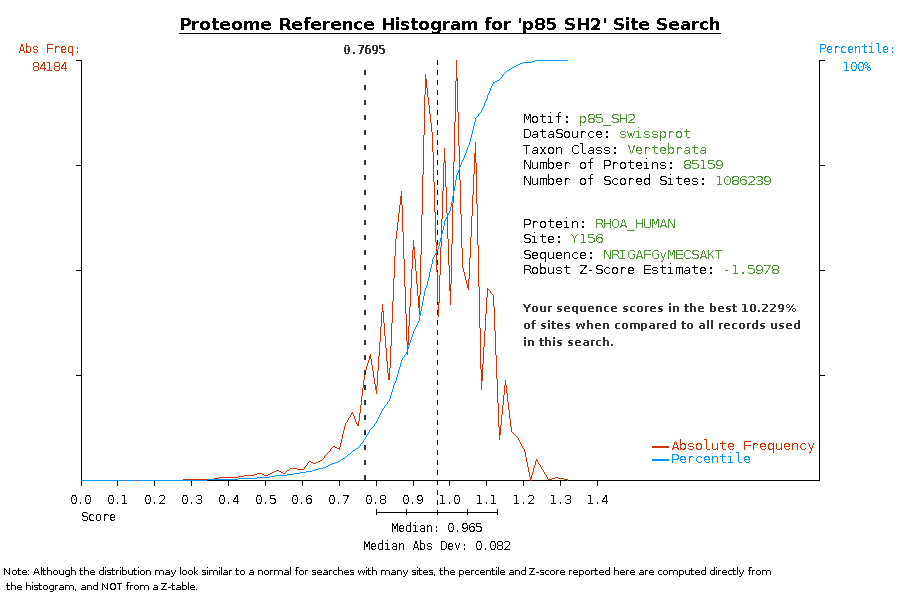

Supplement: Supplementary file 2 — Supplementary Material 2: Supplementary Figure 1 Predicted Interactions of PI3K (p85 SH2 Domain) with AKT1 and RhoA Domains via ScanSite. [file 13008_2025_145_MOESM2_ESM.png]

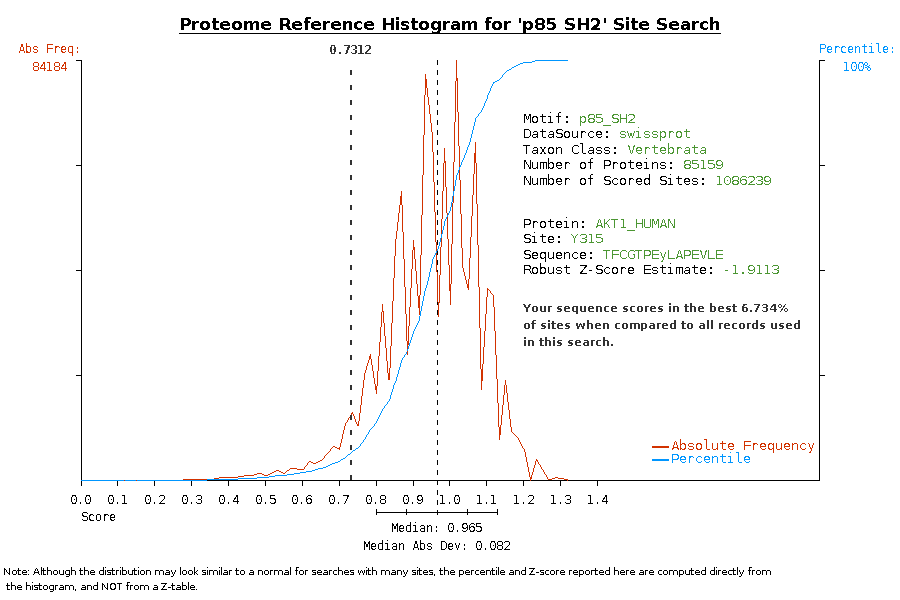

Supplement: Supplementary file 3 — Supplementary Material 3: Supplementary Figure 2 Predicted Interactions of PI3K (p85 SH2 Domain) with AKT1 and RhoA Domains via ScanSite. [file 13008_2025_145_MOESM3_ESM.png]
