# Supplementary material for: Maraviroc enhances Bortezomib sensitivity in multiple myeloma by inhibiting M2 macrophage polarization via PI3K/AKT/RhoA signaling pathway in macrophages
Source: Cell Div. 2025 Feb 14;20:5. doi: 10.1186/s13008-025-00145-1 (PMC11829472; doi:10.1186/s13008-025-00145-1)
Supplement: Supplementary file 4 — Supplementary Material 4: Supplementary Table1: Sequences of all primers. [file 13008_2025_145_MOESM4_ESM.doc]

**Supplementary Table1:** Sequences of all primers.

| **Gene** | **Amplicon(bp)** | **Resources** |
| --- | --- | --- |
| **PI3K** |  |  |
| Forward 5′-CTGCCTGCGACAGATGAGTGATG-3′ | 143 | Primer Express |
| Reverse 5′-ACTGCCCTATCCTCCGATTACCAAG-3′ |
| **AKT** |  |  |
| Forward 5′-CAGGAGGAGGAGGAGATGGACTTC-3′ | 134 | Primer Express |
| Reverse 5′-CCCAGCAGCTTCAGGTACTCAAAC-3′ |
| **RhoA** |  |  |
| Forward 5′-GGTGGATGGAAAGCAGGTAGAGTTG-3′ | 97 | Primer Express |
| Reverse 5′-AACATCGGTATCTGGGTAGGAGAGG-3′ |
| **CCR5** |  |  |
| Forword5′-ATGTGAAGCAAATCGCAGCC-3′ | - | Primer Express |
| Reverse 5′-GCCAGGTTGAGCAGGTAGAT-3′ |
| **CCL3** |  |  |
| Forword 5′-GAGCAGGAAGACTGGCACTT-3′ | - | Primer Express |
| Reverse 5′-TGCCTTCCAGTCACTTGGTC-3′ |
| **CD163** |  |  |
| Forword 5′-TCGCTCATCCCGTCAGTCATCC -3′ | 117 | Primer Express |
| Reverse 5′-GCAAGCCGCTGTCTCTGTCTTC -3′ |
| **β⁃actin** |  |  |
| Forword 5′-TGGCACCCAGCACAATGAA -3′ | 186 | Primer Express |
| Reverse 5′-CTAAGTCATAGTCCGCCTAGAAGCA-3′ |
