# Supplementary material for: Maraviroc enhances Bortezomib sensitivity in multiple myeloma by inhibiting M2 macrophage polarization via PI3K/AKT/RhoA signaling pathway in macrophages
Source: Cell Div. 2025 Feb 14;20:5. doi: 10.1186/s13008-025-00145-1 (PMC11829472; doi:10.1186/s13008-025-00145-1)
Supplement: Supplementary file 5 — Supplementary Material 5: Supplementary Table2: Information of antibody characteristics. [file 13008_2025_145_MOESM5_ESM.doc]

**Supplementary Table2:** Information of antibody characteristics.

| **Name of Antibodies** | **Dilution** | **manufacture** | **Protein Size** |
| --- | --- | --- | --- |
| beta Actin Ab | 1:5000 | Affinity Biosciences(AF7018) | 42kd |
| p-PI3K p85 alpha（Tyr607） Ab | 1:2000 | Affinity Biosciences(AF3241) | 84kd |
| p-RhoA（Ser 188） Ab | 1:2000 | Affinity Biosciences(AF3352) | 22kd |
| p-Pan-AKT1/2/3（Ser 473）Ab | 1:2000 | Affinity Biosciences(AF0016) | 56kd |
